# Long-term recovery from acute cold shock in *Caenorhabditis elegans*

**DOI:** 10.1186/s12860-015-0079-z

**Published:** 2016-01-12

**Authors:** Joseph D. Robinson, Jennifer R. Powell

**Affiliations:** Department of Biology, Gettysburg College, Gettysburg, PA 17325 USA; Present address: Department of Molecular and Cell Biology, University of California Berkeley, Berkeley, CA 94702 USA

**Keywords:** Cold shock, Recovery, *Caenorhabditis elegans*, FSHR-1

## Abstract

**Background:**

Animals are exposed to a wide range of environmental stresses that can cause potentially fatal cellular damage. The ability to survive the period of stress as well as to repair any damage incurred is essential for fitness. Exposure to 2 °C for 24 h or longer is rapidly fatal to the nematode *Caenorhabditis elegans*, but the process of recovery from a shorter, initially non-lethal, cold shock is poorly understood.

**Results:**

We report that cold shock of less than 12-hour duration does not initially kill *C. elegans*, but these worms experience a progression of devastating phenotypes over the next 96 h that correlate with their eventual fate: successful recovery from the cold shock and survival, or failure to recover and death. Cold-shocked worms experience a marked loss of pigmentation, decrease in the size of their intestine and gonads, and disruption to the vulva. Those worms who will successfully recover from the cold shock regain their pigmentation and much of the integrity of their intestine and gonads. Those who will die do so with a distinct phenotype from worms dying during or immediately following cold shock, suggesting independent mechanisms. Worms lacking the G-protein coupled receptor FSHR-1 are resistant to acute death from longer cold shocks, and are more successful in their recovery from shorter sub-lethal cold shocks.

**Conclusions:**

We have defined two distinct phases of death associated with cold shock and described a progression of phenotypes that accompanies the course of recovery from that cold shock. The G-protein coupled receptor FSHR-1 antagonizes these novel processes of damage and recovery.

**Electronic supplementary material:**

The online version of this article (doi:10.1186/s12860-015-0079-z) contains supplementary material, which is available to authorized users.

## Background

All organisms are exposed to a wide variety of stresses during their lives. The ability of an individual to resist and respond to these stresses is crucial for its survival and fitness. One of the most important and constantly changing environmental stresses that an organism must cope with is that of temperature. The direct effects of acute temperature stress have been studied in *Caenorhabditis elegans* and other organisms; however, we have little understanding of the long-term recovery process following exposure to an acute temperature stress.

In *C. elegans*, reproduction is one process affected by heat stress. Worms subjected to a 24-hour heat stress (28–31 °C) survive but exhibit damage to their germline and somatic gonad [1]. Over the next 72 h, many worms recover reproductive fecundity, indicating that the ability to repair damage and recover from stress is an important determinant of fitness. Surprisingly, worms shocked at 31 °C are better able to recover than worms shocked at 29 °C [1], suggesting that the response to stress is both highly sensitive to slight variations in the environment and a complex process of balanced trade-offs.

The long-term recovery from cold stress is less well understood than recovery from heat stress. In both *Drosophila melanogaster* and *Saccharomyces cerevisiae*, a collection of genes is induced during cold shock, but most changes in gene expression occur after return to normal temperatures [2, 3]. This confirms that recovery after the stress is a critical process that is genetically controlled. In *D. melanogaster*, a common measurement of recovery from cold shock is the time required to emerge from the chill coma, a process that usually occurs during the first hour after return to normal growth temperatures [4, 5]. Following this initial rapid recovery, flies often experience a severe defect in mobility; the flies can recover from this mobility defect if the initial cold shock was not too severe [4].

Moderately cold temperatures can be advantageous for *C. elegans*, although extreme cold is detrimental. Worms subjected to mild cold stress (15 °C) have increased longevity; this effect is regulated by several proteins, including the co-chaperone p23, a thermosensitive TRP Channel (TRPA-1), and a fat storage regulating protein (TMEM-135) [6–8]. In contrast, severe cold shock (2–4 °C) is lethal to worms. Loss of insulin signaling increases survival as measured 1 h following acute cold shock [9]. When wild-type worms are acclimatized to 15 °C for as little as 6 h, they have higher survival 1 h following acute cold shock (2–4°) relative to control worms grown at 20 °C. Worms that are acclimatized to 25 °C have decreased survival 1 h after cold shock compared to those grown at 20 °C [9]. These data suggest worms can preemptively alter their physiology to resist damage caused by typically lethal cold exposure. However, we do not yet understand the process by which worms subsequently repair and recover from damage that does occur upon exposure to extreme cold.

*C. elegans* and other organisms can repair and recover from other types of stress. For example, upon oxidative stress, the Nrf transcription factor SKN-1 mediates a detoxification program to mitigate damage caused by reactive oxygen species (ROS) [10–12]. Infection by multiple bacterial pathogens causes *C. elegans* to intentionally upregulate production of ROS as a defense mechanism [13]. To protect from collateral damage, worms coordinately trigger the SKN-1-mediated ROS detoxification program [14]. Interestingly, the G-protein coupled receptor FSHR-1 is required for the survival of exogenous oxidative stress and pathogen stress (infection). Infected *fshr-1* null mutants fail to activate this ROS detoxification program, suggesting that FSHR-1 might be one link between the repair and recovery of oxidative stress and pathogen stress [15].

In this work, we examine the long-term recovery of *C. elegans* after acute cold shock, and define two distinct phases of death that can result. We describe a progression of phenotypes that occur following cold shock, including massive disruption of internal organs. Specifically, we correlate an initial loss and subsequent return of pigmentation with successful recovery from and long-term survival of that cold shock. We also identify the G-protein coupled receptor FSHR-1 as antagonistic to the worm’s ability to recover from cold shock. Our findings highlight the importance not only of surviving severe environmental stresses, but also of successfully repairing damage caused by those stresses.

## Results and discussion

### *C. elegans* has decreased longevity after short cold shock

**Fig. 1:**
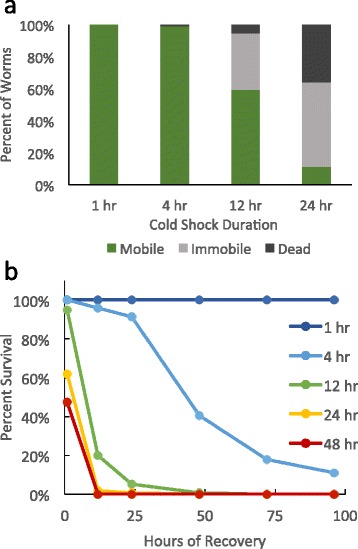
Cold shock reduces longevity after initial recovery. **a** Characterization of the phenotypes of worms after 1 h of recovery at 20 °C following 2 °C cold shocks of different durations. Phenotype was determined by nose touch test. Those worms that only responded with small twitches of the nose or pharynx pumps without any motion of the body were classed as immobile. **b** Percent survival of worms during the 4 days following return to 20 °C after different durations of 2 °C cold shock. Sample sizes are as follows: 1-hour cold shock *N* = 158; 4-hour cold shock *N* = 166; 12-hour cold shock *N* = 483; 24-hour cold shock *N* = 150; 48-hour cold shock *N* = 91

*C. elegans* subjected to acute cold shock of 2 °C for 24–48 h die during or immediately after this exposure to extreme cold (Fig. 1). One hour following a 24-hour cold shock, only 10 % of worms have regained any mobility; the remaining 90 % are either dead or immobile and dying (Fig. 1a). Within 12 h of a 24-hour cold shock, virtually all worms are dead (Fig. 1b).

To assess the longer-term effects of cold, we exposed worms to shorter-duration cold shocks that were not initially lethal. With a 2 °C cold shock duration of 12 h or less, almost all worms survive the initial cold shock (Fig. 1a). However, over the next 96 h, wild-type worms that have been cold shocked for 4 or 12 h have dramatically reduced survival (Fig. 1b). One hour after a 12-hour cold shock, 95 % of worms are alive, though 35 % are immobile; however, fewer than 2 % are alive 24 h later. Despite the fact that virtually all worms are not only alive but most are also mobile 1 h after a 4-hour cold shock, even these relatively healthy-looking worms have dramatically reduced longevity. By 96 h after a 4-hour cold shock, only 11 % of worms survive (Fig. 1a, b).

### Pigmentation of worms decreases after cold shock

Because worms were not killed immediately by shorter (i.e., 4 or 12 h) cold shocks, we hypothesized that the acute exposure to cold caused tissue damage that was not immediately fatal but that could result in delayed lethality if that damage were sufficiently extensive as to be irreparable. To begin characterizing the process of post-cold-shock recovery, we examined the phenotypes of worms following 4-hour or 12-hour 2 °C cold shock.

**Fig. 2:**
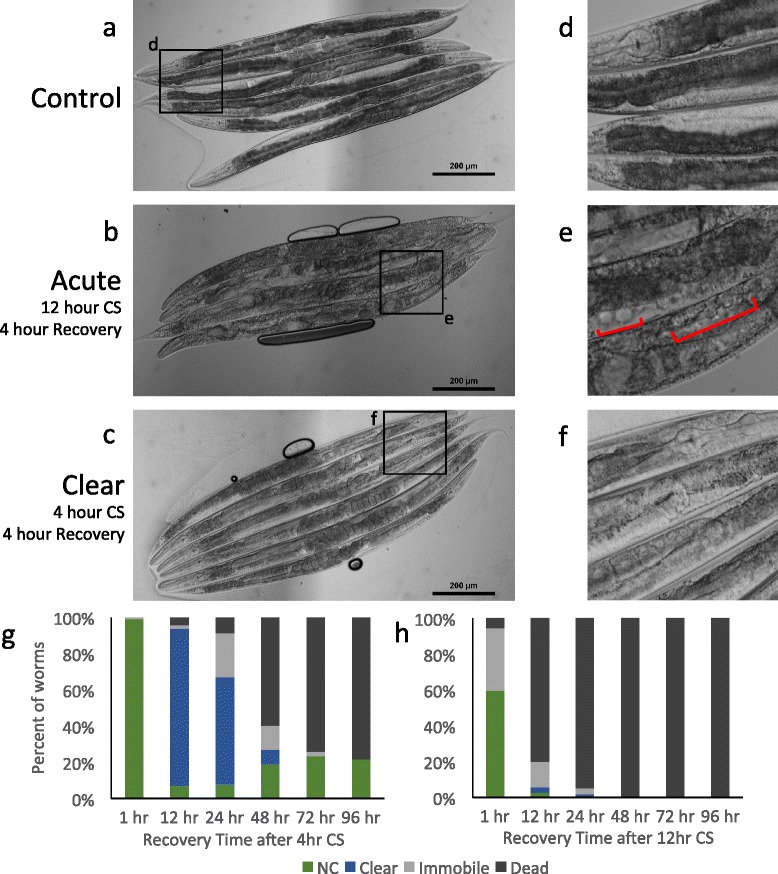
Cold shock produces visually distinct phenotypes within 4 h of recovery. **a**–**c** Examples of the three major phenotypic classes of living worms, with insets **d**–**f**. **a**, **d** Non-clear (NC) worms have dark pigmentation throughout the length of their intestines. **b**, **e** Immobile worms show negligible motion in response to nose-touch, and often contain apparently empty air bubbles. **e** Brackets indicate several of these large bubbles. **c**, **f** Clear worms show reduced pigmentation in their intestines, particularly in the posterior end. Panels **g** and **h** show the percentage of worms exhibiting each phenotype over the course of recovery from 4 h **g** (*N* = 166) and 12 h **h** (*N* = 483) cold shocks

The most dramatic visible phenotype during the recovery from a 4-hour cold shock is a rapid reduction in pigmentation. The intestines of control worms are darkly pigmented due to the presence of birefringent organelles and storage granules (Fig. 2a, d) [16]. Cold shocked worms begin to lose pigmentation within 4 h of recovery at 20 °C, and can be classified as “clear” (Fig. 2c, f). After 12 h of recovery from a 4-hour cold shock, almost all of the worms have developed a very strong clear phenotype (Fig. 2g). Interestingly, over time, a fraction of the worms regain pigmentation, and those worms have a higher rate of survival. By 72 h post-4-hour-cold shock, there are no remaining entirely clear worms; all worms have either regained all or most of their pigmentation, or are immobile or dead (Fig. 2g).

In contrast to a 4-hour cold shock, a 12-hour cold-shock results in very few clear worms. After 12 h of recovery from a 12-hour cold shock, the majority of worms are dead. Of the few remaining live worms, some do have the clear phenotype, indicating that this phenotype can occur following a 12-hour cold shock, but most worms die too quickly to develop this loss in pigmentation (Fig. 2h).

### Two distinct phases of death follow cold shock

Although the majority of 4-hour and 12-hour cold shocked worms are alive 1 h after the cold shock, most become immobile and die within a couple days of “recovery” at 20 °C (Fig. 2g, h). Interestingly, not only is the rate of death dependent on the duration of the cold shock, but also the phenotype at death.

Worms that have been subjected to a 12-hour cold shock die within 24 h. The phenotype of these dead worms corresponds to the worms that die during or immediately following longer cold shocks: their internal organs show reduced definition, resulting in a less distinct, “hazy” internal appearance. The immobility phenotype of 12-hour cold shocked worms is also associated with a reduction in organ definition, and typically heralds death within 12 h (Fig. 2b, e).

Many, but not all, of the worms that have been subjected to a 4-hour cold shock also become immobile and die, although most of the death does not occur until 48–72 h post-cold shock. These immobile/dead worms typically are clear and possess many of the other phenotypes associated with recovery from 4-hour cold shock.

Because we see two phases of death and distinct sets of phenotypes associated with each phase, we hypothesize they are caused by different mechanisms. We will refer to the first phase of death that occurs during or shortly after acute cold shock as “acute death.” We suspect acute death is caused by catastrophic tissue damage due to extreme cold. We will refer to the second phase of death that occurs later, after the worms have begun to progress through the stereotypical set of phenotypes during recovery at 20 °C, as simply “phase two death.” We suspect phase two death occurs in worms that initiate programs to recover from the damage sustained during the acute cold shock, but are either unable to heal sufficiently or jeopardize their own homeostasis during the repair process and therefore subsequently die.

### Cold shock recovery phenotypes and time course

In addition to the striking decrease in pigmentation, we noted several distinctive phenotypes affecting multiple tissues in worms recovering from cold shock. The severity of these phenotypes qualitatively correlates with the duration of the cold shock. We observed that cold-shocked worms display reduced fertility, with fewer viable embryos laid on the plates as they recover. Many but not all of the cold-shocked worms have a qualitatively increased pharyngeal pumping rate relative to the control worms.

Some phenotypes correlate with the eventual outcome of cold shock recovery. Most cold-shocked worms who successfully regain pigmentation concurrently develop a protruding vulva phenotype. As other worms approach death, they become immobile, a phenotype in which they respond to nose-touch by twitching the tip of their nose, pumping the pharynx, or occasionally twitching body wall muscles; however, none of these twitches is successfully converted into motion of the worm’s body. Round structures that appear to be air bubbles develop in some particularly severely affected cold-shocked worms (Fig. 2b, e). These bubbles are only found in immobile worms, and are primarily found after a 12-hour cold shock. They occur less frequently in immobile worms following a 4-hour cold shock.

**Fig. 3:**
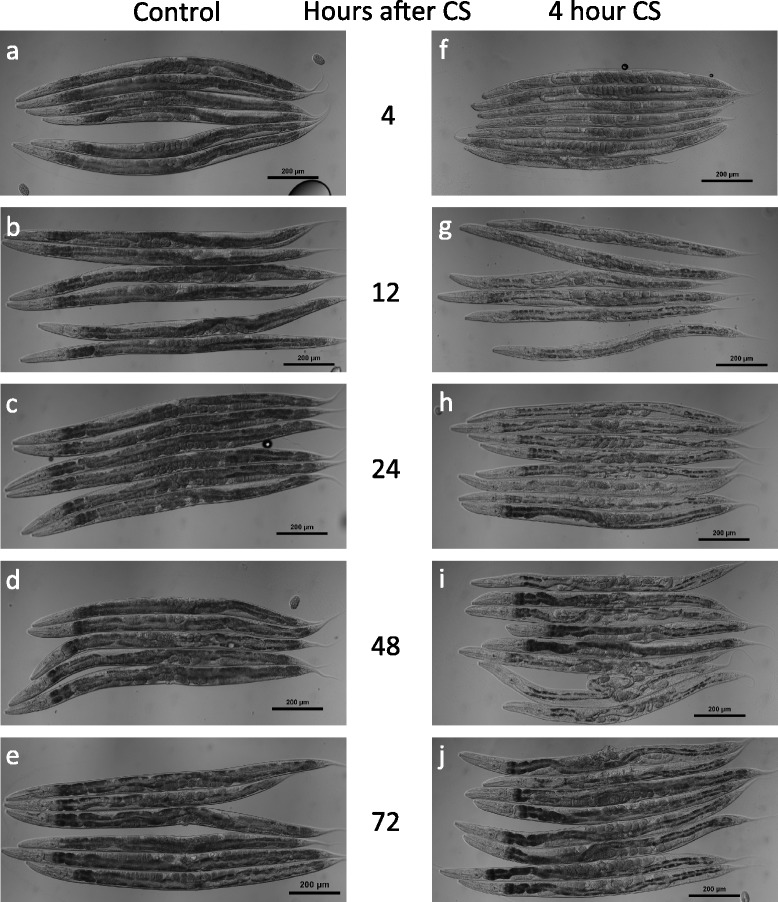
Cold shock recovery progression after 4-hour cold shock. Control **a**–**e** and cold-shocked **f**–**j** worms after 4, 12, 24, 48, and 72 h of recovery, respectively

**Fig. 4:**
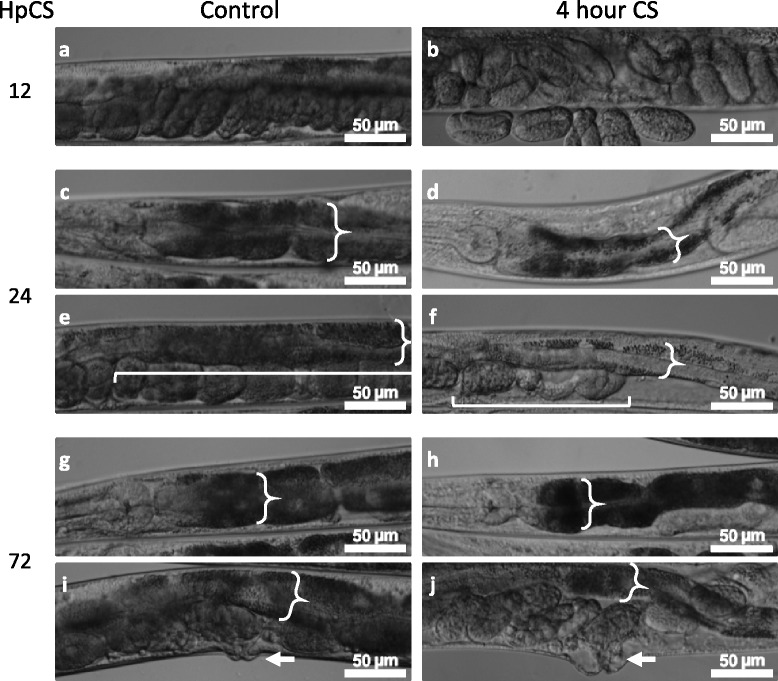
Cold Shock Recovery has diverse phenotypes. **a**–**b** 12 h post 4 h cold shock (HpCS), late stage embryos accumulate in the gonads, eventually causing a bag of worms phenotype. **c**–**f** 24 HpCS the intestine has drastically decreased in width and pigmentation and the gonad arms have shrunk. Braces indicate intestine width; brackets indicate the posterior gonad arm. **g**–**j** Cold-shocked worms that survive to 72 h largely regain their intestinal pigmentation and size; however, they exhibit a protruding vulva phenotype. Braces indicate intestine; arrow indicates vulva

The 4-hour cold shock provided the clearest examples of the variety of phenotypes observed during cold shock recovery, so we examined worms using Differential Interference Contrast microscopy over a 72-hour time-course after a 4-hour cold shock. As we noted above (Fig. 2c, f), worms begin to lose pigmentation within 4 h after cold shock (Fig. 3a, f). By 12 h after cold shock, more pigmentation has been lost, and the worm’s internal organs begin to shrink (Fig. 3b, g). In particular, the gonads appear to wither and there is a distinct disruption of the developing oocytes as well as a decrease in their number relative to controls. Adult worms that had already produced a few embryos before the cold shock accumulate additional embryos within the uterus following cold shock. This eventually leads to a Bag of Worms phenotype and death by 24 h post cold shock (Fig. 4a, b, Additional file 1: Figure S1). The intestinal cells have less pigmentation and decreased width along much of the worm’s length. Large gaps begin to appear in the worm’s body cavity, which contribute to the overall appearance of clearing and are likely caused by the shrinking of the worm’s internal organs.

A day after the cold shock, the damage is more pronounced, with even larger gaps in the worm’s bodies (Figs. 3c, h and 4c–f). The intestinal cells are still further reduced in width, and have continued to lose pigmentation, in many cases losing it entirely (Fig. 4d, f). The posterior gonad arm has usually completely collapsed or occasionally ruptured at this point (Figs. 3h and 4f). The anterior arm is often less damaged, though still severely disrupted, ranging from a reduction in size and disruption of developing oocytes to complete collapse (Fig. 3h).

Two days after a 4-hour cold shock, approximately 60 % of the worms are still alive, and the differences between the worms who will successfully complete their recovery and those who will not survive become readily apparent. In the first class of worms, the intestinal cells begin to regain their pigmentation and some of their size, although the gonads of most worms still appear severely damaged and withered (Fig. 3d, i). The worms who are not destined to recover, however, have lost almost all tissue definition and are immobile, only being able to twitch their noses and pharynx but not move their heads or bodies. These worms typically die within the next 12 h.

Three days after cold shock, nearly all live worms have regained much of their pigmentation (Figs. 3e, j and 4g, h). The majority of these worms have a prominent protruding vulva phenotype, and while their gonads are still partially disrupted, they resume production of oocytes and embryos (Fig. 4i, j). Control non-cold-shocked worms contain a large number of unfertilized oocytes and very few embryos, suggesting that by this time point many of them have depleted their stores of self-produced sperm (Fig. 3e). In contrast, cold-shocked worms that have recovered at 20 °C for 72 h contain embryos that appear to be fertilized and viable (Fig. 3j).

### FSHR-1 plays a role in cold shock survival and recovery

To begin dissecting the genetic mechanisms regulating the recovery from cold shock, we examined the involvement of several genes with known roles in other stress response pathways. The G-protein coupled receptor (GPCR) FSHR-1 has been implicated in the survival of heavy metal and oxidative stress, as well as the innate immune response. Worms containing null mutations in *fshr-1* have wild-type longevity under normal growth conditions, but have increased sensitivity to oxidative, heavy metal, and pathogen stresses [15, 17] . Surprisingly these mutant worms are resistant to 2 °C cold shock (Fig. 4).

**Fig. 5:**
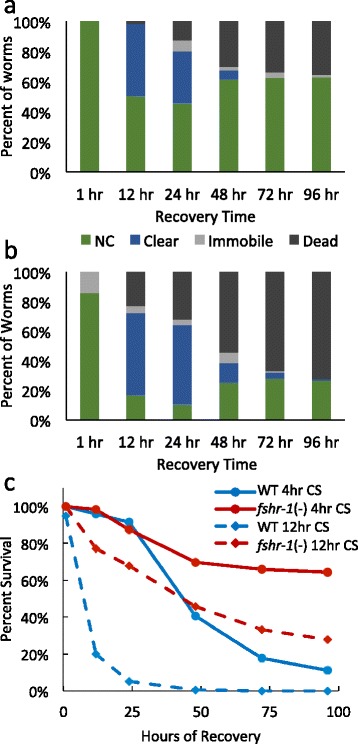
*fshr-1(ok778)* mutants have resistance to and improved recovery from cold shock. Panels **a** and **b** show the percentage of *fshr-1(ok778)* worms exhibiting each phenotype over the course of recovery from **a** 4 h (*N* = 150) and **b** 12 h (*N* = 379) cold shocks. Panel **c** compares the survival curves of *fshr-1(ok778)* worms and wild-type for cold shocks of 4 and 12 h duration. Survival and the distribution of phenotypes were significantly different from WT worms (*P* <0.0001) at all time points, except for the 1 h time point, for both 4 and 12 h cold shocks

As with wild-type worms, all *fshr-1(ok778)* null mutants initially survive a 4-hour acute cold shock (Figs. 2g and 5a, c). Fewer *fshr-1(ok778)* worms than wild-type worms die over the course of a 96-hour recovery at 20 °C. A greater percentage of mutant worms retain their pigmentation during this process and survive. Many mutant worms do become clear during the recovery period. Similar to wild-type worms, those mutant worms who eventually regain their pigmentation, typically beginning by the 48-hour time point, are more likely to survive than those who do not. In both wild-type and mutant worms, very little additional lethality occurs after 72 h of recovery from a 4-hour cold shock. However, the population of wild-type worms stabilizes at approximately 20 % survival, while the population of *fshr-1(ok778)* mutants stabilizes at greater than 60 % survival (Figs. 2g and 4a). There was a significant difference (*p* <0.0001) between the phenotype distributions in wild-type and *fshr-1*(−) worms for all time points except 1 h post cold shock.

Worms lacking *fshr-1* function are also resistant to longer cold shocks. Rather than dying from an acute 12-hour cold shock within 24 h like wild-type worms, *fshr-1(ok778)* mutants behave after a 12-hour cold shock much more like wild-type worms that have only endured a 4-hour cold shock (Figs. 2g, h and 5b, c). All mutant worms are still alive at the end of the cold-shock, and more than 85 % are mobile. The mutants rapidly become clear, but then a portion of them regains pigmentation (Fig. 5b). Worms that retain or regain pigmentation are highly likely to survive the recovery process. Those that die exhibit typical phase two death timing and phenotypes, rather than the acute death experienced by wild-type worms after a 12-hour cold shock. There was a significant difference (*p* <0.0001) between the phenotype distributions in wild-type and *fshr-1*(−) worms for all time points.

Interestingly, *fshr-1(ok778)* mutants show an increase in a bagging phenotype, in which embryos hatch internally before they are laid through the vulva, compared to wild-type worms. Mutants also show a higher than wild-type incidence of extremely rapid nose twitching during the recovery from a 12-hour cold shock.

**Fig. 6:**
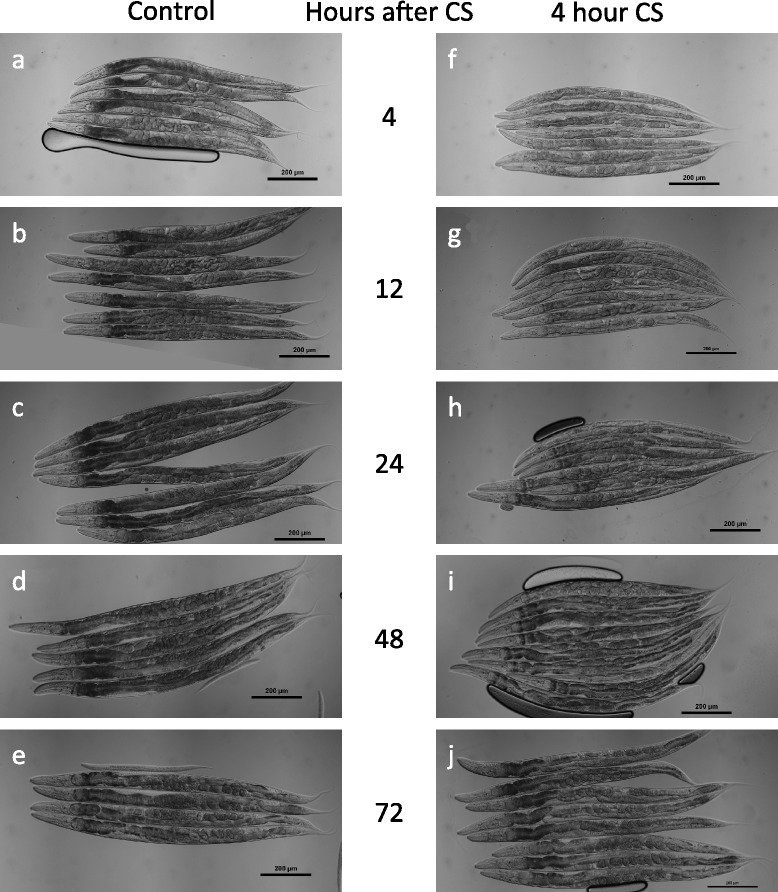
Cold Shock Recovery Progression after 4-hour cold shock for *fshr-1(ok778)* mutant worms. Control **a**–**e** and cold-shocked **f**–**j** *fshr-1(ok778)* worms after 4, 12, 24, 48, and 72 h of recovery, respectively

Closer examination of *fshr-1(ok778)* worms recovering from a 4-hour cold shock reveals disruption to the mutant worms’ internal organs similar to the phenotypes seen in cold-shocked wild-type worms, although in many cases the phenotypes are less severe (Fig. 6). Cold-shocked mutant worms rapidly become clear; however, it is important to note that the non-cold shocked *fshr-1(ok778)* controls have visibly less pigmentation than wild-type worms, especially in the posterior intestine (Fig. 6a, f). Given this reduced basal pigmentation level, the degree to which *fshr-1(ok778)* mutants lose pigmentation is appreciably less than in wild-type. Like wild-type worms, cold-shocked *fshr-1(ok778)* mutants display a reduction in the size of their intestines and some disruption to their gonads, although it does not appear as severe in mutants as in their wild-type counterparts (Fig. 6b, g). Likewise, *fshr-1(ok778)* mutant worms contain fewer embryos following cold shock than non-cold-shocked controls, but this apparent reduction in fertility is less dramatic than the reduction in embryos visible in cold-shocked wild-type worms. Nearly all cold-shocked *fshr-1(ok778)* mutants develop a protruding vulva (Pvul), as do cold-shocked wild-type worms. The combination of this vulval disruption with a higher than wild-type incidence of internal embryos following cold shock of *fshr-1(ok778)* mutants may explain the higher frequency of the Bag of Worms phenotype in these mutants relative to wild-type worms.

## Conclusions

In summary, we have discovered a novel aspect of the *C. elegans* survival of cold shock. Not only must worms survive the acute cold exposure itself, but also they must successfully complete a distinct process of recovery that occurs over several days following the cold shock. The loss of pigmentation and the degradation of the worm’s gonads and intestine over 48 h at 20 °C after an initially non-lethal 2 °C cold shock is evidence that the recovery from cold stress in worms is both a lengthier and more complex process than was previously determined. The GPCR FSHR-1 antagonizes this response because worms lacking this protein are able to recover from a cold shock far more quickly and with a higher frequency than wild-type worms.

We have defined two distinct phases of death associated with cold shock: acute death, which occurs during or immediately following cold shock, and phase two death, which is characterized by a loss of pigmentation and a delayed onset. We propose that acute death is caused by the catastrophic tissue damage caused during longer cold shock, and phase two death results when worms attempt unsuccessfully to repair less severe damage caused by a shorter cold shock. It is possible the delayed onset of phase two death occurs because damage continues to accumulate in improperly functioning cells until it reaches a lethal threshold. Or, the attempts to repair the damage may themselves deplete resources or disrupt the cellular homeostasis to a fatal degree.

Although the long-term effects of cold shock are not well understood, a study in the onion maggot *Delia antiqua* also revealed two different phases of death following cold shock. After exposure to −20 °C, *Delia* larvae die during recovery and display either what the authors term “Type 1” death, in which the larvae first attained adult morphology, or “Type 2” death, in which the larvae did not. The failure to attain adult morphology indicated that Type 2 death was caused by more severe damage. The fraction of larvae that experienced Type 2 death could be increased by suppressing actin polymerization, supporting the author’s hypothesis that acute Type 2 death following severe cold shock is caused by structural damage, while Type 1 death indicates that the larvae were partially able to recover from the cold shock.

Although multiple tissues are affected during the process of cold shock recovery in *C. elegans*, the phenotype of the intestine most closely correlates with a successful outcome to that recovery. Worms en route to acute death do not lose pigmentation. Worms who do become clear might not survive, but they die via phase two death after an apparent attempt at recovery. Initial loss followed by regaining of pigmentation correlates highly with successful recovery from cold shock and long-term survival.

How might the intestine affect the ability of worms to recover from a cold shock? The intestine is a major site of storage for lipids and other key metabolic components and is also, of course, the site of food uptake [16]. It is important to note that increased pharyngeal pumping similar to what we observed in recovering worms is a canonical response to starvation [18]. Additionally, the observed withering and constriction of the gonads in cold shocked worms is very similar to the effects observed in worms that have been starved from L4 into adulthood [19].

We can speculate on two possible models. The dramatic decrease in intestinal size and the reduction of intestinal organelles during recovery from cold shock could be an example of cold-induced damage that causes an inability to absorb nutrients from food and therefore a shortage of critical raw materials needed to repair other damage throughout the worm. If this is the case, phase two death might be caused by an inability to repair damage due to a loss of nutrition.

Alternatively, the clearing and shrinking of the intestine might not be caused by the cold itself, but rather could result from the process of repairing other cold-induced damage. For example, if global metabolism were disrupted by cold, the worm might need to rely on intestinal energy stores to fuel the repair process. This model would suggest that the intestinal clearing occurs as these energy stores become depleted. As worms rapidly use energy for repair, only worms who can restart their metabolism to replenish energy stores or who complete their recovery before energy homeostasis is critically disrupted will survive.

The role of FSHR-1 in this process is intriguing. Mutants lacking *fshr-1* are susceptible to both pathogens and oxidative stressors, have a wild-type response to heat shock, and are resistant to cold shock [15, 17]. In contrast, in most organisms, including *Drosophila*, *Arabidopsis,* and *Saccharomyces*, cold hardiness is associated with increased oxidative stress resistance or the upregulation of antioxidants during cold shock recovery [20–22]. While there are known connections between each of the three stresses that FSHR-1 impacts, the fact that *fshr-1(ok778)* mutants are sensitive to pathogen and oxidative stress but resistant to cold stress seems paradoxical. It is thus unlikely that *fshr-1*′s role in cold stress is directly related to its role in oxidative stress or immunity. Given that *fshr-1(ok778)* mutants have less intestinal pigmentation, it is tempting to speculate that the connection between FSHR-1 and cold shock recovery may relate to metabolism or energy homeostasis. Additional dissection of the role this receptor plays in the recovery from cold shock will likely illuminate the mechanisms by which *C. elegans* integrates the various stress responses required for survival in complex environments.

## Methods

### *C. elegans* growth and maintenance

N2 wild-type (Bristol) and AU28 *fshr-1(ok778)* hermaphrodites were maintained at 20 °C on Nematode Growth Medium (NGM) seeded with *Escherichia coli* OP50, as previously described [23]. To obtain young adult worms, L4 larvae were picked to fresh seeded NGM plates and allowed to develop at 20 °C for 12–14 h. Worms were visually observed, and any gravid or larval worms removed. These plates were then transferred to 2 °C for cold shock assays.

### Cold shock recovery assays

Cold shock assays were performed by transferring seeded NGM plates containing approximately 30 healthy young adult worms to 2 °C for 1, 4, 12, 24, or 48 h. During the cold-shock period, the door to the incubator was not opened to ensure temperature consistency. At the end of the cold-shock period, worms were removed and transferred to 20 °C to recover. The recovery phenotypes and/or viability were scored 1, 4, 12, 24, 48, 72, and 96 h after return to 20 °C. Scoring could not be performed immediately upon return to 20 °C, or while still at 2 °C, because when *C. elegans* is exposed to cold temperatures it enters a cold coma that precludes any motion. The hour delay between return to 20 °C and scoring is to permit the worms to awaken from the cold coma.

At each time point, worms were scored and sorted to separate seeded 3.5 cm NGM plates according to the phenotypes observed. Death was defined as a nose touch producing no response. Immobile was defined as a nose touch producing only small nose twitches, pharyngeal pumping, or minor twitching motion of muscles inside the worm that did not translate to any motion of the body. Mobile worms, i.e. worms that actively moved their head or body when touched on the nose, were separated into clear and non-clear by a qualitative measure of the degree of pigmentation loss. Generally, worms with less pigmentation than age-matched control worms throughout the length of the intestine were defined as clear. Worms that either had normal pigmentation or patchy coloration in which some sections of the worm were equivalent to controls were defined as non-clear.

### Cold shock imaging

Young adult hermaphrodite worms were cold shocked as described above and then shifted to 20 °C to recover. At the indicated time points, worms were picked to slides containing a drop of M9 buffer with 5 mM sodium azide on a 2 % agarose pad and aligned using a pick as they became paralyzed. They were then imaged using a Nikon 90i DIC microscope. Control worms were treated identically except for the lack of cold shock. At least 20 worms were photographed for each category. Exposure times were held constant across all conditions, and images were not manipulated other than rotation and cropping.

### Ethics statement

No vertebrate animals were used for these studies and no ethical approval was required.

## Availability of supporting data

Raw data are available in LabArchives, LLC (DOI: 10.6070/H4GM85BK).

## Additional file

Additional file 1: Figure S1. Cold Shock associated Bag of Worms phenotype. Wild-type worms that died between 12 and 24 h after a 4-hour cold shock exhibit dramatically reduced pigmentation. These worms exhibit the Bag of Worms phenotype, defined by the presence of internally hatched larvae. (PDF 421 kb)

## Abbreviations

GPCR: G-protein coupled receptor
NGM: nematode growth media
CS: cold shock
