# Supplementary figures and images for: Long-term recovery from acute cold shock in Caenorhabditis elegans
Source: BMC Cell Biol. 2016 Jan 12;17:2. doi: 10.1186/s12860-015-0079-z (PMC4709947; doi:10.1186/s12860-015-0079-z)

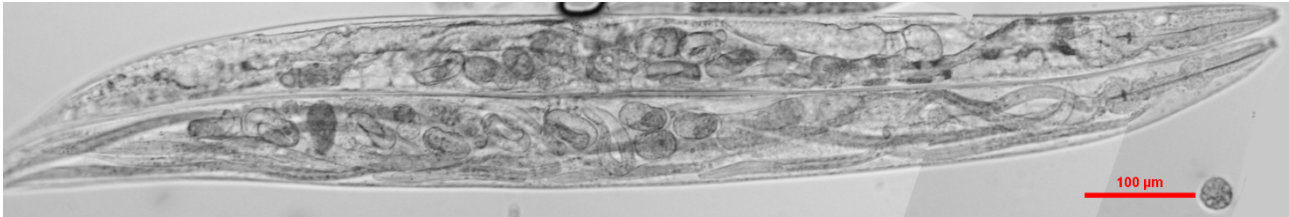

Supplement: Additional file 1: Figure S1. — Cold Shock associated Bag of Worms phenotype. Wild-type worms that died between 12 and 24 h after a 4-hour cold shock exhibit dramatically reduced pigmentation. These worms exhibit the Bag of Worms phenotype, defined by the presence of internally hatched larvae. (PDF 421 kb) [file 12860_2015_79_MOESM1_ESM.pdf]
